# Scent detection dogs as a novel method for oestrus detection in an endangered species, the Tasmanian devil (*Sarcophilus harrisii*)

**DOI:** 10.3389/fvets.2023.1224172

**Published:** 2023-10-17

**Authors:** Hannah E. Roberts, Kerry V. Fanson, Naomi Hodgens, Marissa L. Parrott, Pauleen Bennett, La Toya Jamieson

**Affiliations:** ^1^Wildlife Conservation and Reproductive Endocrinology Lab, School of Agriculture, Biomedicine and Environment, La Trobe University, Melbourne, VIC, Australia; ^2^Wildlife Detection Dog Program, Wildlife Conservation & Science, Zoos Victoria, Melbourne, VIC, Australia; ^3^Wildlife Conservation & Science, Zoos Victoria, Parkville, VIC, Australia; ^4^Anthrozoology Research Group, Department of Psychology, Counselling and Therapy, La Trobe University, Bendigo, VIC, Australia

**Keywords:** captive breeding, conservation, non-invasive, endangered wildlife, Dasyurid, marsupial, olfactory, reproductive state

## Abstract

Captive breeding is a critical tool for conservation of endangered species. Identifying the correct time to pair males and females can be a major challenge for captive breeding programmes, with current methods often being invasive or slow. Detection dogs may provide a non-invasive way to determine female receptivity, but this has not been explored in captive wildlife. This exploratory study investigated the use of detection dogs as a novel method of oestrus detection in the endangered Tasmanian devil (*Sarcophilus harrisii*). Faecal samples were collected from 11 captive female devils during the breeding seasons of 2020 and 2021. Three dogs with prior detection experience were trained and subsequently assessed (*n* = 188 searches per dog), on their ability to discriminate between oestrus and non-oestrus devil faecal samples, in a one sample set-up. When assessed on training samples, dogs were able to correctly discriminate oestrus from non-oestrus with a mean sensitivity of 69.1% and mean specificity of 65.7%. When assessed on novel samples, their sensitivity to oestrus dropped (mean sensitivity of 48.6%). However, they were still able to correctly identify non-oestrus samples (mean specificity of 68.1%). This study is the first to explore detection dogs’ ability to identify oestrus in a captive breeding programme for endangered wildlife, providing a promising tool for non-invasive monitoring of reproductive status in wildlife.

## 1. Introduction

Conservation of species is of growing concern as the world’s biodiversity declines, in what some describe as the sixth mass extinction (1, 2). Conservation efforts frequently focus on *in situ* strategies that aim to protect a species in their natural habitat (3). However, *ex situ* conservation programmes, such as captive breeding, can also be crucial in protecting a species from extinction. Captive breeding can help conserve a species by maintaining insurance populations, managing low genetic diversity and providing individuals for wild release (4, 5). However, captive breeding of endangered species can be challenging, especially in species where relatively little is known about their reproduction (6, 7).

Effective captive breeding management hinges on accurate identification of an individual’s reproductive status (7, 8). The period of oestrus – when females are receptive to mating – is especially important to identify accurately, to know when to pair males and females to maximise reproductive success, when to avoid pairings if mating is not wanted, and when to perform assisted reproductive techniques. The window of oestrus can be brief. When males and females are housed separately, introducing them at the wrong time (when the female is not receptive) can not only be unsuccessful, but sometimes dangerous (7). Identification of oestrus must often be conducted with minimal disturbance or stress to the animal, especially for endangered species; thus, animal managers need a method that is non-invasive, as well as accurate and timely (6–8).

Possible oestrus detection methods for use in endangered species include faecal or plasma hormone analysis, vaginal smears, and behavioural monitoring, but each has limitations (6–9). While faecal hormone analysis is non-invasive, it is costly and can take several days to obtain results, which could result in the period of receptivity being missed (10–12). In comparison, plasma sampling provides a real-time snapshot of circulating hormone levels, but similarly takes time to get results, with sample collection being invasive (8–12). Vaginal cytology, as well as examination of pouch morphology in marsupials, can be accurate methods of oestrus detection, but both are similarly invasive (9, 13, 14). Behavioural changes are non-invasive, but may be variable (15, 16), and are subtle and time consuming to monitor, especially in cryptic, shy, or nocturnal species (9). Given the importance of accurately identifying receptivity for successful production of young in captivity, an accurate, quick, and non-invasive oestrus detection method would be invaluable.

With their excellent olfactory ability, scent detection dogs present a novel technique for potentially identifying oestrus in captive wildlife. Scent detection dogs are increasingly employed in medical settings to detect various biological odours and metabolic states [e.g. cancer of the lungs, bladder, and skin (17–21); hypoglycaemia (18, 22); seizures (18, 23); and even COVID-19 (24, 25)], as well as for conservation projects (18, 26). Prior studies have found that dogs can successfully detect oestrus in dairy cows, with sensitivity varying from 57.6 to 80.3%, depending on sample type and training/assessment methods (27–30). As the hormonal changes that occur during oestrus are similar across all mammals, and many species communicate reproductive status via pheromones (31), it is likely that dog detection of oestrus could be also applicable to captive wildlife species. Only one detection dog study has explored reproductive state in captive wildlife (pregnancy in polar bears, *Ursus maritima*), with the dog excelling in training but failing to adapt to new variations of the target scent (32). Reproductive states – like pregnancy or oestrus – likely have complex odour profiles that are of unknown composition, and may vary between individuals and throughout their cycle. Therefore, to properly examine detection performance, it is crucial to examine dogs’ ability to generalise to samples that they have not encountered during training (33).

The endangered Tasmanian devil (*S. harrisii*) provides an ideal species to examine the feasibility of dog detection of oestrus. Since 1996, devil numbers have declined by more than 80% due to habitat destruction and the ongoing spread of Tasmanian devil facial tumour disease (DFTD), a contagious and deadly cancer (34–36). Thus, captive breeding is critical for their continued survival. Captive breeding of devils has had success, but faces ongoing challenges. Due to their cryptic, occasionally aggressive nature, a hands-off approach to captive management is best for devil welfare (37). Thus, invasive methods of oestrus detection [such as plasma hormone analysis, vaginal cytology, or examination of the pouch (38–40)] are unsuitable. Comparatively, faecal hormone analysis is non-invasive, but, even ignoring transport time to a lab, the preparation and analysis of devil faecal samples takes a minimum of 3 days (40). For a species with such a short period of receptivity – with oestrus lasting on average 10 days, and keepers preferring to pair within the first three – this delay is impractical. Additionally, although there are known changes in faecal hormones in the devil during oestrus (38, 40), as is the case with other marsupials these changes do not necessarily provide a precise prediction of oestrus in advance (13). Most commonly, devil keepers instead monitor for changes in lethargy, inappetence, and the formation of a fatty neck roll on the back of the female’s neck (which males grasp and use to drag the female around during mating) (37, 40), but these signs of oestrus appear to be becoming increasingly subtle in captivity, and are very time-consuming to monitor (M. Parrott and M. Zabinskas, 2020, pers. Comm.).

Dog detection of oestrus may be particularly successful for Tasmanian devils because devils communicate with conspecifics by scent marking at latrines, where they deposit urine, faeces and anal secretions (41, 42). Devils are able to distinguish between different individuals via scent, and both male and female devils spend longer investigating female faeces (42), supporting the idea that scent marking may help communicate female reproductive status. In captivity, keepers can also sometimes predict the receptivity of females by observing the response of males to the female’s scent when they are housed nearby (37). Faecal samples can be collected non-invasively as part of a normal husbandry routine, allowing access to scent samples without excess labour. Considering the importance of olfactory communication in devils and the proven olfactory ability of dogs, it seems likely that scent detection dogs could be a viable method of oestrus detection in devils.

This exploratory study is the first to examine the feasibility of employing oestrus detection dogs to assist the captive breeding of wildlife. Two key objectives are addressed: (1) to determine if dogs can be trained to discriminate between oestrus and non-oestrus odours in Tasmanian devil faecal samples; (2) to test the ability of the dogs to generalise oestrous scents from training samples to novel samples. These will be the first steps towards exploring the employment of oestrus detection dogs to improve the reproductive management and captive breeding of not only devils, but other endangered wildlife.

## 2. Methods

### 2.1. Overview

This work was conducted with Healesville Sanctuary, Zoos Victoria, as part of their Tasmanian devil captive breeding programme. Ethics approval for the use of the devils and the detection dogs was granted via Zoos Victoria’s Animal Ethics Committee (permit ZV20007), with the project approved by the Tasmanian Department of Natural Resources and Environment/Zoo and Aquarium Association Tasmanian Devil Captive Research Advisory Group. As is described below, all dog training was flexible, tailored to the individual, and was constantly reassessed based on the dogs’ welfare and performance, to allow them the best chance for success.

### 2.2. Tasmanian devil faecal samples

#### 2.2.1. Study population

This study included 11 captive-born female devils of breeding age (2 to 4 years old) housed at Healesville Sanctuary. Individuals were housed separately, unless paired for breeding. Devils had access to two types of dens (a concrete den and a log mound) and a variety of natural structures, including climbing logs and native plants. Devils were fed a starve-gorge diet (varying in both content and quantity) to mimic natural diets, except during the breeding season where they were fed daily to allow keepers to identify changes in appetite, and to best support females when they had young. Water was available *ad libitum*. Over the course of this study, seven of the 11 females were paired with males, with six (two in 2020; four in 2021) successfully producing young.

#### 2.2.2. Oestrus determination

Mating receptivity of female devils was estimated by staff at Healesville Sanctuary via changes in activity levels and decrease in food consumption (37), as per current Sanctuary protocols. Seven females were subsequently paired with genetically suitable males and monitored, with pairs separated if prolonged aggression was observed. Parturition was identified via observation of a decrease in appetite, and the occurrence of birthing postures [a tripod stance with visible abdominal contractions (37)]. Because un-mated Tasmanian devils undergo pseudopregnancy, whereby they display all the signs of pregnancy but lack an embryo (43), the start of oestrus could be confirmed by back-dating from the birthing or pseudo-birthing date by 25 days, and corroborating with behavioural and appetite observations.

#### 2.2.3. Sample collection

Faecal samples were collected from female devils during February–July of 2020 and 2021. Keepers collected fresh samples during normal husbandry rounds, which were conducted 1–2 times/day. Most samples were estimated to be a maximum of 6–8 h old. Mean monthly temperatures in the area during the period of collection ranged from minimums of 3.6–13.2°C, to maximums of 13.4–26.0°C (Bureau of Meteorology, Coldstream^1^). Samples were collected via an inverted zip-lock bag to avoid contamination and placed in individual sterile airtight containers. Each faecal sample was later divided into one to six separate glass specimen jars (depending on faeces size), to minimise the risk of losing an entire sample if inadvertently contaminated, and stored at −20°C. Samples were not collected when females were paired during oestrus for several reasons: (1) females’ food consumption decreased during oestrus, resulting in less faecal production, (2) keeper interaction was reduced to avoid disruption to mating devils, and (3) keepers could not confidently distinguish between male or female scat when devils were paired.

For this study, ‘target’ (oestrous) samples were limited to the first 8 days of oestrus, to capture the period in which keepers prefer to pair devils. ‘Non-target’ (non-oestrous) samples were limited to the 11 days immediately before the start of oestrus, in order to refine the dogs’ level of discrimination. In total, 47 oestrus scats and 78 non-oestrus scats were collected and used. Of those, 23 oestrus and 55 non-oestrus samples were used for training (‘familiar samples), and 24 oestrus and 23 non-oestrus samples were only used during the assessment phase of the study (‘novel’ samples).

### 2.3. Research dogs

Three healthy, desexed dogs (two male, one female) were trained for this study (Table 1). All three dogs are part of Zoos Victoria’s Wildlife Detection Dog Programme. Two of the dogs (Kip and Daisy) had previously been involved in a short-term, preliminary investigation into dog detection of devil oestrus prior to this study. All dogs had extensive prior experience on scent detection projects and were chosen for their highly motivated temperaments.

**Table 1:** Details of detection dogs trained in the study.

Name	Breed	Age (yrs)	Sex	Desexed	Involved in preliminary investigation
Kip	Australian Kelpie mix	7	Male	Yes	Yes
Daisy	Lagotto Romagnolo	5	Female	Yes	Yes
Moss	Labrador Retriever	3	Male	Yes	No

### 2.4. Detection dog training

Detection dog training was conducted indoors, 1 day a week, from February to July of 2022. Each training day comprised 2–3 training sessions (approximately 15 min each per dog). An assistant was responsible for placing samples on a scent wheel and would face away from the wheel during the search, to avoid giving behavioural cues that could bias the dog or handler. Training was reward based, using both food and toy rewards, and using a clicker as a conditioned reinforcer. Training was flexible and adjusted based on the dog’s individual requirements, with consideration of practicality for real-world application. All training sessions were recorded via video camera (Go Pro, Hero9 Black), with each dog’s true positives, false positives, true negatives, false negatives, and samples used being documented.

When in use, each specimen jar (without lid) containing devil faeces was placed in a separate metal scent pot with a mesh lid to allow dogs to sniff, but not access the source of, the scent. To avoid saliva contamination that could influence the dog’s choice during training, scent pots were sanitised between each dog using 70% isopropanol wipes and allowed to dry before use (44, 45), and 2–5 used scent pots were also randomly replaced with clean, unused pots. During assessments, however, clean scent pots were used for each dog. After each training/assessment day, scent pots were washed in a designated dishwasher (with no detergent) to remove organic material, air-dried, and then sprayed with 70% isopropanol and left to dry (44, 45).

#### 2.4.1. Discrimination training

Training started by presenting one oestrus sample to the dog and rewarding the dog for smelling and showing interest in the target. This was followed by training an alert behaviour (sitting) at the target sample. When the dog was confidently alerting, discrimination training commenced, in which 1–3 pots were presented to the dog on the scent wheel, comprising one oestrus sample and 0–2 non-oestrus samples. The dog was again rewarded for sniffing the target pot, and this then progressed to rewarding when the dog correctly alerted to the target. Once dogs were locating the correct sample with confidence, all searches were conducted with the handler blind to the location of the target sample to avoid unintentional body language that could bias the dog (46). The dog’s alert behaviour for each search was confirmed verbally with the assistant so that the dog could be rewarded if correct. Dogs progressed to the one-pot yes/no training (see below) when they were confident in locating the oestrus sample with the handler blind to its location. This was achieved in approximately 2–3 months, with each dog completing at least eight discrimination training days.

#### 2.4.2. Yes/no set-up

Training ultimately progressed to a yes/no set-up, which allows for simpler statistical analysis and provides a constant probability of chance (47). This would also be the most practical set-up if used for ongoing oestrus monitoring in the captive breeding programme. In the yes/no set-up, only one sample is presented on the scent wheel and the dog performs separate alerts for a target (yes) or non-target (no) sample (48, 49).

**Figure 1:**
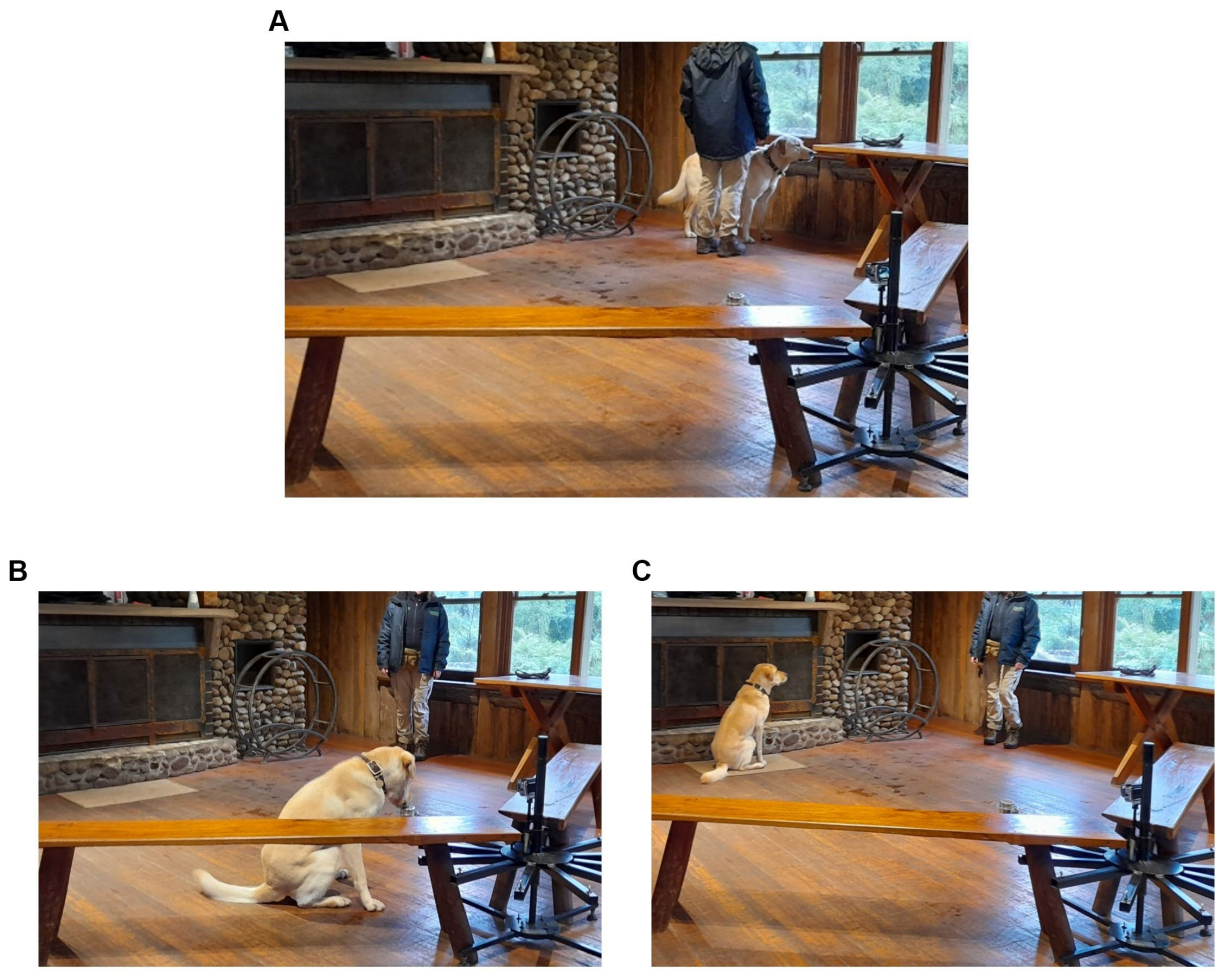
The yes/no set-up. **(A)** Dogs begin with the handler to the side of the set-up. Only one sample is presented to the dog on the scent wheel, with a designated mat 2–3 metres away. Wooden benches provide a barrier to funnel the dog towards the pot and prevent searching around the wheel. **(B)** Example of a correct target (oestrus) indication. **(C)** Example of a correct non-target (non-oestrus) indication.

For the yes/no searches, the dog and handler were stationed on the side until the dog was cued to search (Figure 1A). If an oestrus sample was present, the dog was trained to alert by sitting at the pot (Figure 1B). For non-oestrus samples, the dog was trained to sit at a designated mat 2–3 m away (Figure 1C). After either being rewarded for a correct alert or called away for a wrong alert, the dog returned to the handler to prepare for another search. This separate alert for non-target samples, and the ensuing reward for a correct answer, is deemed important as dogs may be more comfortable providing a designated separate behaviour for non-target scents, instead of performing no standard behaviour (47). Yes/no training was conducted over 3–4 months, with each dog completing 13–19 yes/no training days in that time.

### 2.5. Detection dog assessments

Assessments were conducted indoors using the yes/no set-up in July and August of 2022. Each dog completed a total of 188 searches over 4–5 non-consecutive days. Each day comprised 2–3 assessment sessions of 20 searches, with a break of approximately 10 min between each session. Assessments were conducted in a double-blind manner. The handler was blind to the type and order of samples; and the assistant, after placing each sample, moved out of sight of the dog-handler team into an adjacent corridor for the duration of the search. Once the handler determined a target or non-target sample based on the dog’s alert behaviour, the sample type was confirmed verbally with the assistant so the dog could be rewarded if correct. As in training, assessments were video recorded and true positives, false positives, true negatives and false negatives were noted for each dog.

**Figure 2:**
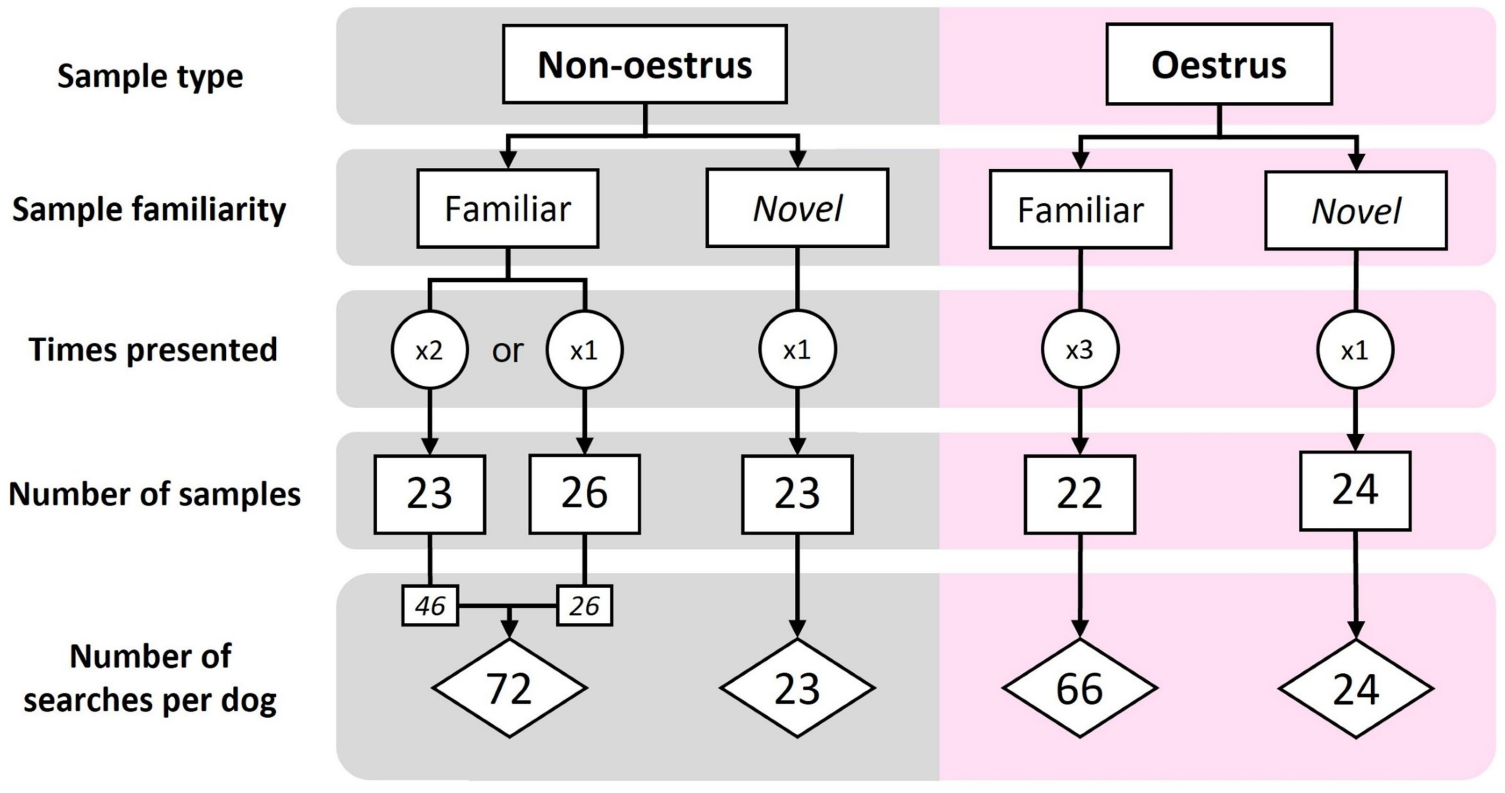
The sample make-up and number of searches for the familiar and novel assessments. Each familiar sample was presented to each dog 1–3 times, while novel samples were presented only once per dog.

Samples were classified as ‘novel’ (new to the dog) or ‘familiar’ (used in training). During each assessment session, the searches included a mix of oestrus and non-oestrus samples (sample type), as well as familiar and novel samples (sample familiarity). Order and type of samples was pseudo-randomised using an online number randomisation service [Random.org^2^ (RRID:SCR_008544)], according to the following criteria. Each session contained 8–12 oestrus and 8–12 non-oestrus samples. To avoid undue dog frustration, only five novel samples were presented during each assessment session (a ratio of 3:1, familiar to novel samples), with no more than five of each sample type presented consecutively. Thus, each familiar sample was presented to each dog multiple times over the course of the assessments (three times for oestrus, 1–2 times for non-oestrus; Figure 2). All novel samples were presented to the dog only once (Figure 2). In total, dogs were assessed on 71 familiar samples and 47 novel samples, amounting to 141 familiar searches and 47 novel searches per dog (Figure 2).

#### 2.5.1. Data analysis

Data were analysed in R (Version 1.4.1106). Level of significance was set at *p* < 0.05 for all statistical tests.

**Figure 3:**
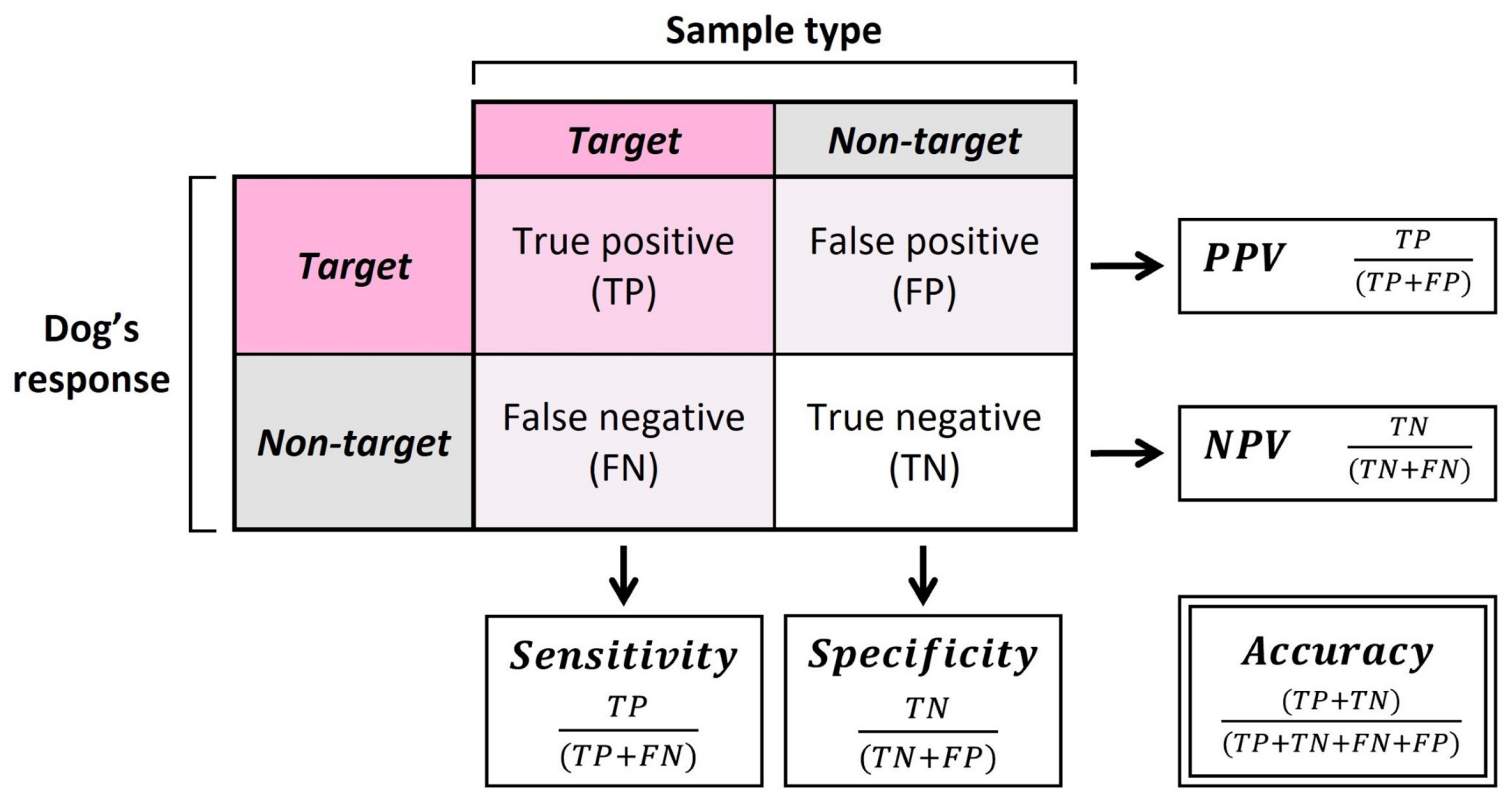
Calculations of sensitivity, specificity, positive predictive value (PPV), negative predictive value (NPV), and accuracy.

To assess the detection abilities of the dogs during the assessments, five main summary statistics were calculated: the accuracy, sensitivity, specificity, positive predictive value (PPV), and negative predictive value (NPV; Figure 3). Accuracy was calculated as the overall probability of a correct sample identification. Sensitivity and specificity were calculated as the probability that the sample was identified correctly for a given sample type (oestrus or non-oestrus). PPV and NPV were calculated as the probability that the alert was correct for a given alert behaviour (oestrus or non-oestrus). Exact binomial tests (function binom.test) were run to compare dog performance against the rate of chance (50%).

To further examine how different factors affected overall dog performance, we ran a generalised linear model with binomial distribution (function glmer). Dog performance for each search was scored as 1 (correct alert) or 0 (incorrect alert). The predictor variables included in the model were sample type (oestrus/non-oestrus), sample familiarity (familiar/novel), the sample type x familiarity interaction, breeding outcome, and oestrus day. We subsequently ran a post-hoc pairwise comparison with a Tukey correction. Breeding outcome for each devil was classified as follows: ‘not paired’ = females were not housed with a male; ‘not pregnant’ = females were paired with a male for mating but did not produce pouch young; ‘pregnant’ = females were paired with a male and successfully produced pouch young. Oestrus day was included to examine whether dog performance (probability of correct identification) changed throughout the sampling period; if there are gradual changes in odour cues around the onset of oestrus, this may affect dog performance. Oestrus day was calculated as the difference between a sample’s collection date and the start of oestrus, with the first day of oestrus set as Day 0. Dog ID was included as a random effect to control for repeated searches by each dog.

## 3. Results

### 3.1. Training success

**Figure 4:**
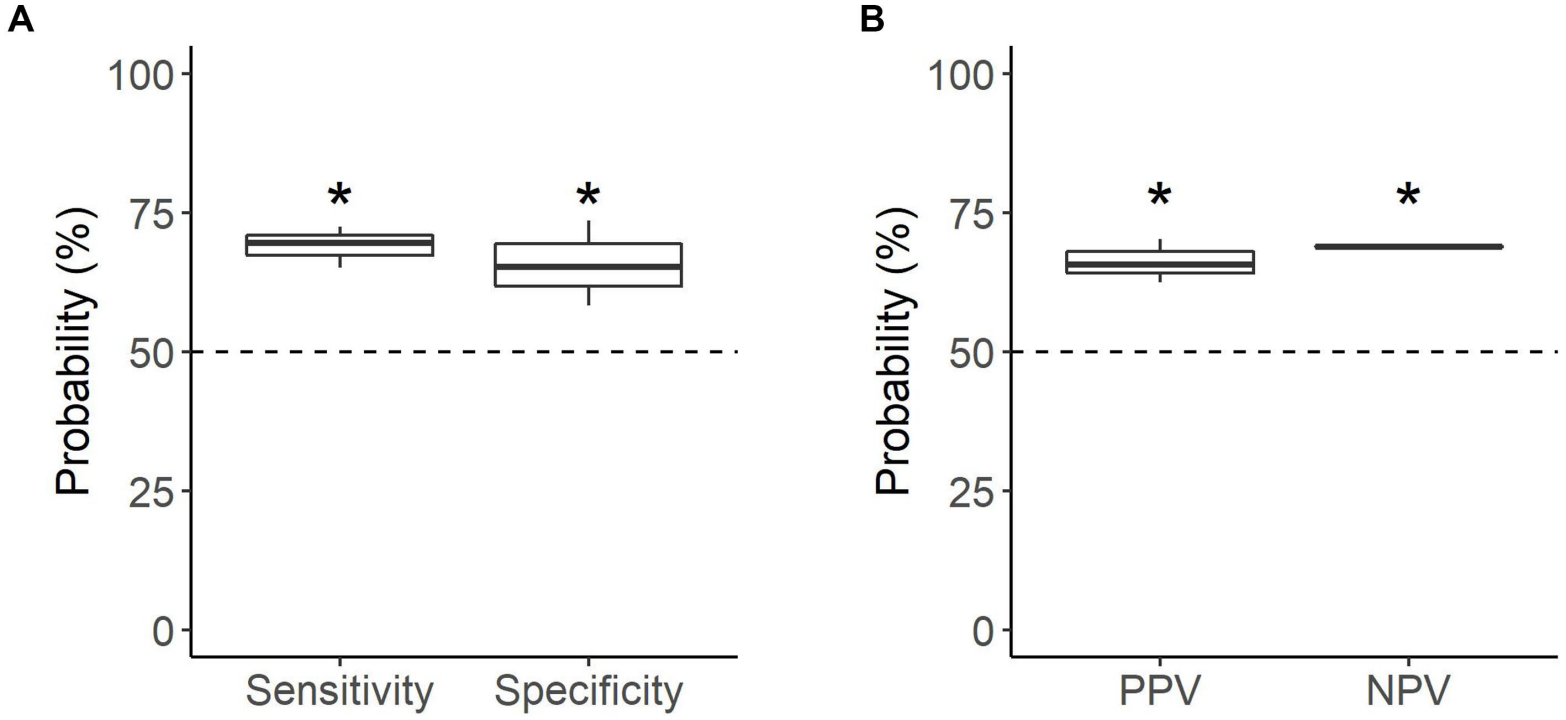
Performance of the detection dogs during the familiar assessments. **(A)** Probability that sample type was correctly identified (mean sensitivity and specificity). **(B)** Probability that dog’s alert type was correct (mean PPV and NPV). Dotted line = rate of chance (50%). * = significantly different from rate of chance (*p* < 0.05).

All dogs were able to correctly identify familiar samples (*n* = 141 searches) significantly better than chance, with a mean accuracy of 67.4% (*p* < 0.001; Table 2). Dogs were equally successful at identifying oestrus and non-oestrus samples, achieving a mean sensitivity of 69.1% and mean specificity of 65.7% on familiar samples (*p* < 0.001; Figure 4A). Similarly, the dogs’ positive and negative alerts were both more likely to be correct than chance (*p* < 0.001; Figure 4B).

**Table 2:** Oestrus detection ability of each dog during the familiar assessments: accuracy, sensitivity, specificity, positive predictive value (PPV), negative predictive value (NPV), and probability of the dog providing an oestrus alert.

	Daisy	Kip	Moss	Mean	CI (95%)
Accuracy	65.2	69.5	67.4	67.4 ± 1.2	62.7–71.8
Sensitivity	72.5	65.2	69.5	69.1 ± 2.1	62.3–75.3
Specificity	58.3	73.6	65.3	65.7 ± 4.4	59.0–72.0
PPV	62.5	70.3	65.8	66.1 ± 2.3	59.2–72.2
NPV	68.9	68.8	69.1	68.9 ± 0.09	62.1–75.2
Probability of oestrus alert	56.7	45.4	51.8	51.3 ± 3.3	46.4–56.2

Values are expressed as a percentage, with means expressed as mean ± SEM (standard error of the mean).

Individually, all the dogs were able to identify samples above the rate of chance. Detection of familiar, non-oestrus samples had the greatest disparity in performance, with specificity ranging from 58.3 to 73.6% (Table 2). Of the dogs, Kip’s oestrus alerts were the most reliable (PPV of 68.9%), with Daisy’s the least reliable (PPV of 62.5%; Table 2). Conversely, the reliability of the dogs’ non-oestrus alerts differed minimally, with NPV ranging from 68.8 to 69.1% (Table 2). Regardless of performance, Daisy and Moss had a higher proportion of performing the oestrus alert, whereas Kip tended towards the non-oestrus alert (Table 2).

### 3.2. Generalisation

**Figure 5:**
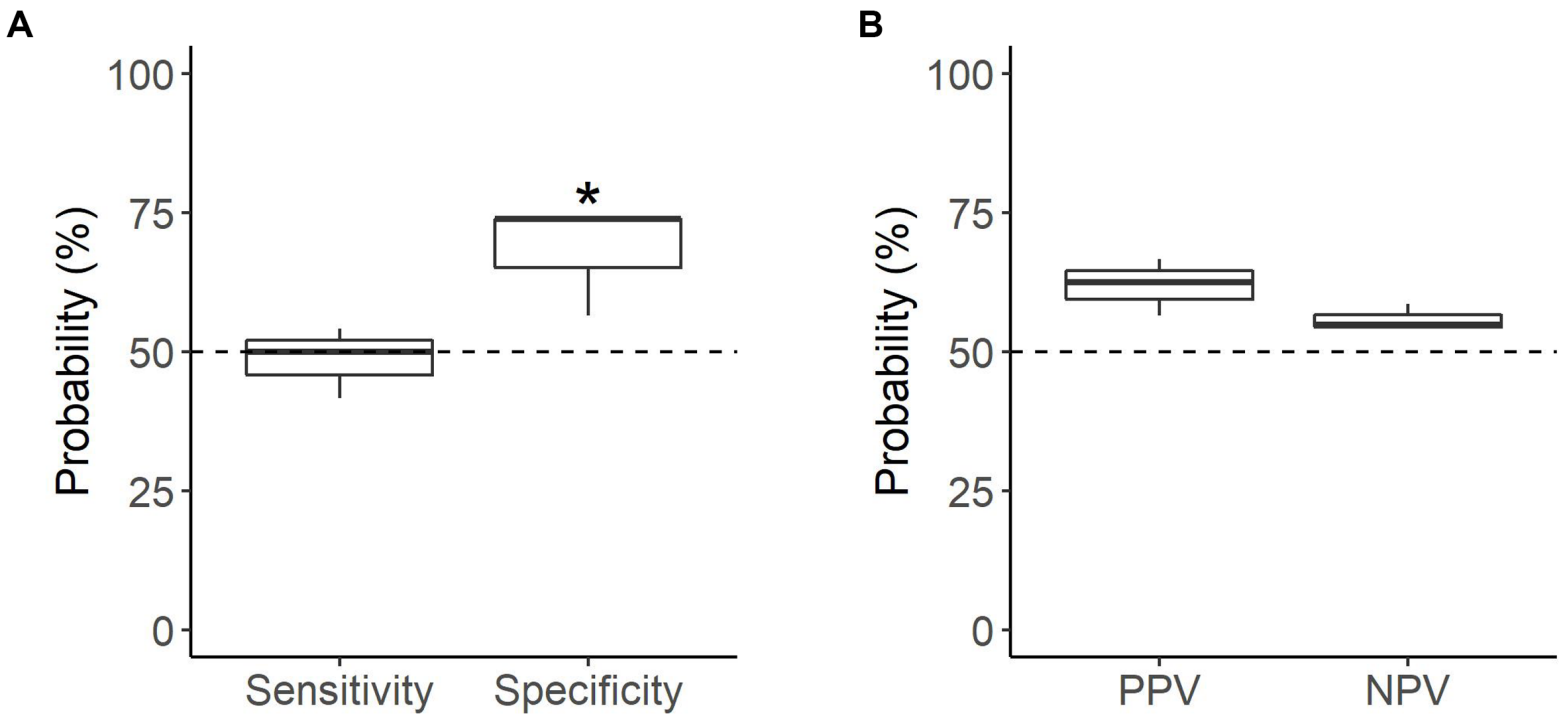
Performance of the detection dogs during the novel assessments. **(A)** Probability that sample type was correctly identified (mean sensitivity and specificity). **(B)** Probability that dog’s alert type was correct (mean PPV and NPV). Dotted line = rate of chance of a correct alert (50%). * = significantly different from rate of chance (*p* < 0.05).

When tested on novel samples (*n* = 47 searches), the dogs were able to correctly identify samples only marginally better than chance, with a mean accuracy of 58.2% (*p* = 0.064; Table 3). Performance in the novel assessments depended on sample type; dogs were able to correctly identify non-oestrus samples better than chance, with a mean specificity of 68.1% (*p* = 0.0035), but were not able to similarly identify oestrus samples, with a mean sensitivity of 48.6% (*p* = 0.91; Figure 5A). Neither oestrus nor non-oestrus alerts were more accurate than chance (*p* = 0.11 and *p* = 0.33, respectively; Figure 5B).

**Table 3:** Oestrus detection ability of each dog during the novel assessments: accuracy, sensitivity, specificity, PPV, NPV, and probability of the dog providing an oestrus alert.

	Daisy	Kip	Moss	Mean	CI (95%)
Accuracy	55.3	57.4	61.7	58.2 ± 1.9	49.6–66.4
Sensitivity	54.2	41.7	50.0	48.6 ± 3.7	36.7–60.7
Specificity	56.5	73.9	73.9	68.1 ± 5.8	55.8–78.8
PPV	56.5	62.5	66.7	61.9 ± 2.9	47.6–74.0
NPV	54.2	54.8	58.6	55.9 ± 1.4	44.7–66.8
Probability of oestrus alert	48.9	34.0	38.3	40.4 ± 4.4	32.3–49.0

Values are expressed as a percentage, with means expressed as mean ± SEM (standard error of the mean).

Individual dog performance varied more widely against novel samples. Similar to the familiar assessments, performance on novel non-oestrus samples differed the most between dogs, ranging from 56.5 to 73.9% specificity (Table 3). Kip struggled to identify oestrus with a sensitivity of 41.7%, while Daisy was the most successful with a sensitivity of 54.2%, barely above chance (Table 3). Overall, Moss’s alerts were the most accurate, although the accuracy of the dogs’ non-oestrus alerts differed minimally, with a range of 54.2 to 58.6% (Table 3). All dogs tended towards the non-oestrus alert, regardless of performance (Table 3).

### 3.3. Longitudinal changes in alerts

**Figure 6:**
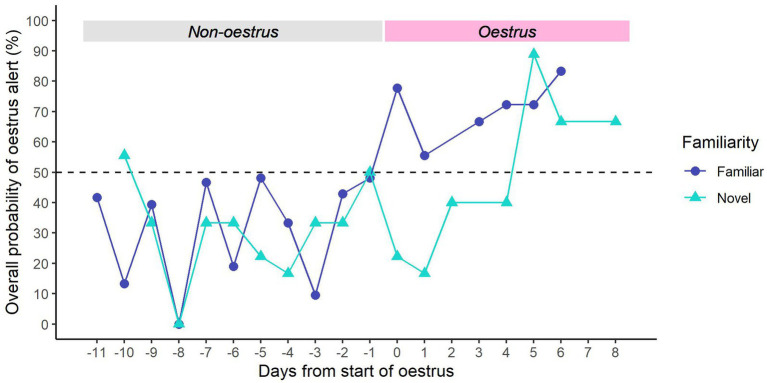
Overall probability of providing an oestrus alert vs. days from the start of oestrus. The first day of oestrus = day 0. Dark blue line (●) = familiar samples; light blue line (▲) = novel samples. Dotted line = rate of chance (50%).

For familiar samples, we found that dogs were consistently more likely to give the ‘non-oestrus’ alert in the days leading up to oestrus, and the ‘oestrus’ alert after the onset of oestrus (Figure 6). There seemed to be a general increase in oestrus alerts across the oestrus window, peaking on Day 6 of oestrus.

For novel samples, dogs were generally more likely to give the ‘non-oestrus’ alert in the days prior to oestrus, except for Day −10 (Figure 6). However, they continued to give the ‘non-oestrus’ alert for the first 4 days of oestrus. From Day 5 of oestrus, dogs were consistently more likely to give the ‘oestrus’ alert.

### 3.4. Predictors of performance

We found a significant interaction between sample type and sample familiarity (χ^2^ = 5.88, *df* = 1, *p* = 0.015). Dogs were less successful at detecting novel oestrus samples compared to other sample types, although this difference was only significant for the familiar oestrus comparison (*z* = 3.14, *p* = 0.0093). None of the other pairwise comparisons were significant.

The devil’s breeding outcome did not significantly affect the dogs’ performance (not paired, not pregnant, or pregnant; χ^2^ = 0.76, *df* = 2, *p* = 0.69). Similarly, there was no significant change in the probability of correct alerts across the sampling period, highlighting that dogs were more likely to give ‘non-oestrus’ alerts prior to oestrus and ‘oestrus’ alerts during oestrus (χ^2^ = 0.82, *df* = 1, *p* = 0.37).

## 4. Discussion

This study is the first to investigate the use of detection dogs to determine oestrus in a wildlife species. While detection dogs have previously been employed for a variety of biological or metabolic states, oestrus detection is still a new area. In this study, dogs were able to discriminate between oestrus and non-oestrus Tasmanian devil faecal samples used in training at a rate greater than predicted by chance, with a mean accuracy of 67.4%. Performance declined with novel oestrus samples, indicating they were not able to generalise an oestrous scent to new samples; however, dogs were able to generalise non-oestrus samples from training and maintain their performance with novel samples. The likelihood of an oestrus alert also increased throughout oestrus for both familiar and novel samples, suggesting some recognition of a changing oestrous scent. Although the number of dogs used in this study was limited, the results of this study are promising for future explorations into oestrus detection in wildlife.

Recent studies into oestrus detection in dairy cows reported varying success, with sensitivity ranging from 57.6 to 80.3% (27–30). Comparatively, this study presented an overall mean sensitivity of 58.9%. Differences in training set-up can make it difficult to compare studies; the aforementioned studies applied the commonly used scent line-up (whereby one target is presented in a row amongst multiple non-targets), while we chose the less common yes/no set-up. Training and assessment set-up can have an important impact on detection success, with each type of search presentation having benefits and challenges (47, 50). One benefit of the yes/no set-up is that, along with faster search turn-over, it provides the ability to reward the dog for each correct search regardless of sample type. This is important for maintaining search motivation, which is a crucial factor that can influence a dog’s performance. Dogs can learn to expect certain sample presentations, and their search persistence and engagement can drop accordingly. For example, repeated searches without successful detection of a target can negatively impact search motivation and cause a decline in performance for subsequent searches (51, 52). Thorough randomisation of the order of novel and familiar samples is important to reduce the likelihood of repeated difficult or incorrect trials and the dog not being rewarded too many times in a row (47, 50). One disadvantage of the yes/no set-up is that dogs may have a bias towards a specific alert behaviour. For example, rats had a left- or right-lever bias in a similar two-response method (53). We found that Daisy and Moss had a higher probability of performing an oestrus alert in the familiar trials, while Kip was more likely perform a non-oestrus alert. Comparatively, in the novel trials, all dogs were more likely to perform the non-oestrus alert. However, it should be noted that bias can also be a problem with other set-ups. For example, scent line-ups can result in dogs being more likely to indicate at the last sample in a row (47). If dog detection of oestrus was to be used operationally in a breeding programme, presenting only one sample to a dog as per the yes/no method is faster and much more practical, as opposed to managing a full line-up or scent wheel (a circular line-up) with multiple non-target samples.

It is also possible that the type of sample used may have impacted detection ability. One uncertainty in this study was the efficacy of using easily collected and non-invasive faecal samples for scent detection of oestrus, as that substrate has not been thoroughly explored in this context. Previous studies determined vaginal fluid to be the most effective training sample type for oestrus detection in cows, compared with saliva, milk and urine (27–30). This is further supported by Sankar and Archunan (54), who found that male mice were most interested in investigating bovine vaginal fluid, followed by saliva, faeces, and milk, respectively. Dogs trained on vaginal fluid were also able to generalise to odours in urine and milk with similar detection success (29). While it is possible that other bodily fluids could provide a better indication of reproductive status for oestrus detection, the invasive collection of vaginal fluid and saliva unfortunately render them non-viable options for oestrus detection in the Tasmanian devil, and likely other endangered species in captive breeding programmes. Comparatively, faeces can be collected daily with minimal keeper-animal interaction, which is crucial for the welfare of the normally shy, solitary devil. With the importance of faecal olfactory signalling in devil reproduction, and the ease of collection in captivity, faecal samples still remain a favourable source of odour for use in devil oestrus detection.

Olfactory generalisation is the ability of a detection dog to extrapolate their training to new variations of a target odour, and it is crucial to a dog’s operational efficacy (33). For example, differences in the diet, illness, medication use, or individual scent of the animal, that affect a sample’s odour, must all be ignored in favour of the common target (in this case, ‘oestrus’). The desired level of generalisation is situation dependant: a low level of generalisation (and therefore high discrimination) may result in missed target samples and a lower sensitivity; whereas a high level of generalisation (and low discrimination) may result in false alerts and a lower specificity (33). In this study, our dogs were able to correctly discriminate between oestrus and non-oestrus samples used in training. However when presented with novel samples, the dogs poorly generalised to the oestrus samples, resulting in a lower sensitivity. Our results are similar to prior attempts to train dogs to detect prostate cancer in humans (55) and pregnancy in polar bears (32), where dogs had a high detection rate during training, but when tested were unable to detect novel samples at a rate above chance. Both studies concluded that the dogs may have memorised the odours of the specific training samples, as opposed to identifying the target’s overall scent profile. This is likely the case in our study, as the range of available training samples was unavoidably limited due to the small number of endangered devils available, the reduced appetite in receptive females, and the limitations in sample collection for paired devils.

Dogs can memorise at least 40 distinct odours and can retain this learning for over 12 months without additional training (56). Therefore, targets with complex or unknown odour profiles (like oestrus, cancer, or pregnancy) likely necessitate a broad range of training samples to accurately convey the target odour’s natural variance (33, 55, 57). The number and variety of samples needed to establish successful generalisation and a high sensitivity likely varies with the odour type and training method. Johnen et al. (47) recommend training on 100 target and non-target samples each for complex odours like cancer (or oestrus), but obtaining such a number of samples from endangered wildlife is often prohibitive. Encouragingly, although our dogs struggled to generalise to oestrus samples, they were able to maintain their performance with novel non-oestrus samples, maintaining a high specificity. In training, we had nearly two times more non-oestrus than oestrus samples, which may have improved the dogs’ familiarity with the non-oestrus scent and thus their generalisation. In the future, having more oestrus samples available for training would minimise repeated exposures that could familiarise a dog to a sample’s specific odour profile. Improving the dogs’ concept of the oestrous scent may also minimise any tendency towards bias in the yes/no method.

During management of Tasmanian devils, a false indicator of oestrus (leading to devils being paired when the female is not receptive) can potentially lead to conflict between animals, and even injury or death (37). A high sensitivity is usually the goal for detection scenarios; but lacking that, a high specificity could alternatively provide enough confidence to be similarly functional. If dogs can reliably determine when devils are *definitely not* in oestrus, this may still help keepers avoid the untimely pairing of unreceptive devils. Alternatively, having multiple dogs assess a single sample could add an extra layer of confidence in alerts, and find a balance between maximising sensitivity or specificity (58). Knowing the individual PPV and NPV of each dog could also assist decision making. For example, keepers may be more confident in the accuracy of a given oestrus alert from a dog with a high PPV, compared with an alert from a dog with a lower PPV. A higher sensitivity and specificity than displayed in this study would be required before canine detection of oestrus is a feasible singular method for devils in captivity. However, dog detection could nonetheless be used as a complementary method to assist keepers, in combination with the current method of behavioural observation of devils.

Many questions remain regarding the feasibility of oestrus detection dogs to determine mating receptivity in Tasmanian devils. In this study, the likelihood of the dogs providing an oestrus alert increased with samples further into oestrus; a change which, although delayed, was still evident in the more difficult novel samples. This suggests that despite the variation in performance, there were odour differences across oestrus that the dogs could detect. Specifically, these results imply that the olfactory cues used by the dogs increase throughout oestrus, leading to more accurate identification of oestrus later in the cycle. Expanding knowledge of the oestrous odour profile and how it changes over the course of the reproductive cycle would greatly assist future research into dog detection of oestrus. Prior studies have identified oestrous-specific volatile organic compounds (VOCs) in a variety of biological excretions from different species, including urine (59–63), vaginal fluid (64), sweat (65) and faeces (66, 67). Given their importance in scent marking, devil faeces presumably contain an oestrous-specific scent that dogs could identify. Examination of the changes in VOCs throughout the reproductive cycle would help validate this assumption and confirm the validity of using faeces as an odour source for dog detection of oestrus. It is unknown to what extent these oestrus-linked compounds are species-specific; it is possible that detection dog training samples could be supplemented with easier-to-collect oestrus samples from a closely-related species (32). Comparing the faecal oestrus VOCs of related species (such as the spotted-tail quoll, *Dasyurus maculatus*, or the fat-tailed dunnart, *Sminthopsis crassicaudata*) with those of the devil would give insight into this possibility. Understanding the cyclical changes in the oestrous odour would aid in exploring the extent to which the dogs are able to identify specific days of female receptivity. In devils, the ideal pairing time for successful production of young is 3 days into oestrus (38, 40). Correct identification of the first three oestrus days could be of immeasurable help to devil keepers struggling to determine receptivity from behaviour or appetite changes, and would ultimately improve breeding prospects.

The main limitations of the study were the limited sample size (that may have hampered generalisation) and the unknown nature of the ‘oestrus’ scent profile. Although we had limited oestrus samples, we still found a significant effect in novel non-oestrus and all familiar samples. A greater number of training samples would lessen the risk of memorisation for specific samples, and we expect the dogs would be better able to generalise an oestrus scent profile across individuals. The specific chemical molecules that indicate ‘oestrus’ in devil faecal samples are yet unknown, and it is also possible that detection of oestrus may have been affected by changes in the samples over time during storage and use. Identifying the specific chemical signature of oestrus would allow us to characterise changes in the scent profile throughout oestrus, develop more targeted training techniques (e.g., synthetic scents), and monitor degradation over time. Improving the breadth of knowledge around the ‘oestrous odour’ and its changes over time would be of great benefit to future studies into oestrus detection and could help inform future training modification.

## 5. Conclusion

Accurate identification of oestrus is crucial to successful captive breeding of wildlife, but current methods are often inaccurate, slow or invasive. This study explored the potential of detection dogs to identify oestrus in the faeces of an endangered wildlife species, the Tasmanian devil. Three detection dogs were able to successfully and consistently identify non-oestrus samples, but struggled to generalise an oestrous scent to new samples. This approach requires further research to confirm its validity and improve its efficacy; in the future, the viability of this approach would be further improved by increasing the number and variety of samples available for training, to refine dogs’ generalisation to new scents. Research into the unknown nature of the oestrous odour profile would help validate faeces as an appropriate odour source and illuminate how odour changes throughout the reproductive cycle. Pinpointing the most relevant window of the oestrous period would also improve the utility of this technique for management of breeding programmes. The use of oestrus detection dogs could ultimately improve current devil breeding protocols, welfare, and reproductive success in captivity. With further research, this approach could also be expanded to captive breeding programmes of other endangered species to improve conservation efforts worldwide.

## Data availability statement

The original contributions presented in the study are publicly available. This data can be found here: Open At La Trobe (OPAL) https://opal.latrobe.edu.au/, under the DOI 10.26181/22883564.

## Ethics statement

The animal study was approved by Zoos Victoria’s Animal Ethics Committee (permit ZV20007), with the project approved by the Tasmanian Department of Natural Resources and Environment/Zoo and Aquarium Association Tasmanian Devil Captive Research Advisory Group. The study was conducted in accordance with the local legislation and institutional requirements.

## Author contributions

HR, LJ, NH, PB, and KF contributed to experimental design of the study. HR, LJ, and NH performed the experiments. HR curated the data and wrote the first draft of the manuscript. HR, LJ, and KF contributed to the statistical analyses. MP and PB provided scientific input and edited the manuscript. All authors contributed to the article and approved the submitted version.

## Funding

This research was undertaken as part of a Master of Science degree for which HR received a La Trobe Research Training Program stipend scholarship.

## Footnotes

^1^ http://www.bom.gov.au/climate/averages/tables/cw_086383.shtml

^2^ https://www.random.org/sequences/

## References

[1] Proença V Pereira HM. Comparing extinction rates: past, present, and future In: Levin SA, editor. Encyclopedia of biodiversity. Second ed. Waltham: Academic Press (2013). 167–76.

[2] Ceballos G Ehrlich PR Barnosky AD Garcia A Pringle RM Palmer TM. Accelerated modern human-induced species losses: entering the sixth mass extinction. Sci Adv. (2015) 1:e1400253. doi: 10.1126/sciadv.1400253, PMID: 26601195 PMC4640606

[3] Pritchard DJ Fa JE Oldfield S Harrop SR. Bring the captive closer to the wild: redefining the role of ex situ conservation. Oryx. (2012) 46:18–23. doi: 10.1017/s0030605310001766

[4] Leus K. Captive breeding and conservation. Zool Middle East. (2011) 54:151–8. doi: 10.1080/09397140.2011.10648906

[5] Bowkett AE. Recent captive-breeding proposals and the return of the ark concept to global species conservation. Conserv Biol. (2009) 23:773–6. doi: 10.1111/j.1523-1739.2008.01157.x, PMID: 19220367

[6] Wildt DE Wemmer C. Sex and wildlife: the role of reproductive science in conservation. Biodivers Conserv. (1999) 8:965–76. doi: 10.1023/a:1008813532763

[7] Holt WV Pickard AR Rodger JC Wildt DE. Reproductive science and integrated conservation. Cambridge, United Kingdom: Cambridge University Press (2002).

[8] Brown JL. Comparative ovarian function and reproductive monitoring of endangered mammals. Theriogenology. (2018) 109:2–13. doi: 10.1016/j.theriogenology.2017.12.004, PMID: 29249329

[9] Alexandre RS Nei M Alexsandra FP Gislayne CXP Keilla MM Lívia BC. Estrus cycle monitoring in wild mammals: challenges and perspectives In: Rita Payan C, editor. Theriogenology. Rijeka: IntechOpen (2017). 2.

[10] Wielebnowski N Watters J. Applying fecal endocrine monitoring to conservation and behavior studies of wild mammals: important considerations and preliminary tests. Israel J Ecol Evol. (2007) 53:439–60. doi: 10.1560/ijee.53.3.439

[11] Kersey DC Dehnhard M. The use of noninvasive and minimally invasive methods in endocrinology for threatened mammalian species conservation. Gen Comp Endocrinol. (2014) 203:296–306. doi: 10.1016/j.ygcen.2014.04.022 24798579

[12] Pukazhenthi BS Wildt DE. Which reproductive technologies are most relevant to studying, managing and conserving wildlife? Reprod Fertil Dev. (2004) 16:33–46. doi: 10.10371/rd03076 14972101

[13] Witt RR Rodger JC. Recent advances in tools and technologies for monitoring and controlling ovarian activity in marsupials. Theriogenology. (2018) 109:58–69. doi: 10.1016/j.theriogenology.2017.12.006, PMID: 29254685

[14] Andrews CJ Thomas DG Welch MV Yapura J Potter MA. Monitoring ovarian function and detecting pregnancy in felids: a review. Theriogenology. (2020) 157:245–53. doi: 10.1016/j.theriogenology.2020.06.036, PMID: 32818882

[15] Lindburg DG Czekala NM Swaisgood RR. Hormonal and behavioral relationships during estrus in the giant panda. Zoo Biol. (2001) 20:537–43. doi: 10.1002/zoo.10027

[16] Swinbourne AM Phillips CJC Janssen T Lisle A Keeley T Johnston SD. Reproductive biology of captive female southern hairy-nosed wombats (*Lasiorhinus latifrons*). Part 2: oestrous behaviour. Reprod Fertil Dev. (2018) 30:1424–33. doi: 10.1071/rd17539, PMID: 29742384

[17] Bijland LR Bomers MK Smulders YM. Smelling the diagnosis: a review on the use of scent in diagnosing disease. Neth J Med. (2013) 71:300–7. Available at: https://pubmed.ncbi.nlm.nih.gov/23956311/ PMID: 23956311

[18] Browne C Stafford K Fordham R. The use of scent-detection dogs. Ir Vet J. (2006) 59:97. Available at: https://www.researchgate.net/publication/261663456_The_use_of_scent-detection_dogs

[19] Ehmann R Boedeker E Friedrich U Sagert J Dippon J Friedel G. Canine scent detection in the diagnosis of lung cancer: revisiting a puzzling phenomenon. Eur Respir J. (2012) 39:669–76. doi: 10.1183/09031936.00051711, PMID: 21852337

[20] Hayes JE McGreevy PD Forbes SL Laing G Stuetz RM. Critical review of dog detection and the influences of physiology, training, and analytical methodologies. Talanta. (2018) 185:499–512. doi: 10.1016/j.talanta.2018.04.010, PMID: 29759233

[21] Willis CM Church SM Guest CM Cook WA McCarthy N Bransbury AJ. Olfactory detection of human bladder cancer by dogs: proof of principle study. British Med J. (2004) 329:712–714A. doi: 10.1136/bmj.329.7468.712, PMID: 15388612 PMC518893

[22] Rooney NJ Morant S Guest C. Investigation into the value of trained glycaemia alert dogs to clients with type I diabetes. PloS One. (2013) 8:e69921. doi: 10.1371/journal.pone.0069921, PMID: 23950905 PMC3737201

[23] Catala A Grandgeorge M Schaff J-L Cousillas H Hausberger M Cattet J. Dogs demonstrate the existence of an epileptic seizure odour in humans. Sci Rep. (2019) 9:4103. doi: 10.1038/s41598-019-40721-4, PMID: 30923326 PMC6438971

[24] Eskandari E Shiri M Aliyazdi H Farahani RH Nezami-Asl A Laripour R. Sniffer dogs as a screening/diagnostic tool for COVID-19, a proof of concept study. BMC Infect Dis. (2020) 21:243. doi: 10.1186/s12879-021-05939-6 PMC7934999 33673823

[25] Jendrny P Schulz C Twele F Meller S von Köckritz-Blickwede M Osterhaus ADME. Scent dog identification of samples from COVID-19 patients – a pilot study. BMC Infect Dis. (2020) 20:536. doi: 10.1186/s12879-020-05281-3, PMID: 32703188 PMC7376324

[26] Paula J Leal MC Silva MJ Mascarenhas R Costa H Mascarenhas M. Dogs as a tool to improve bird-strike mortality estimates at wind farms. J Nat Conserv. (2011) 19:202–8. doi: 10.1016/j.jnc.2011.01.002

[27] Fischer-Tenhagen C Johnen D Le Danvic C Gatien J Salvetti P Tenhagen BA. Validation of bovine oestrous-specific synthetic molecules with trained scent dogs; similarities between natural and synthetic oestrous smell. Reprod Domest Anim. (2015) 50:7–12. doi: 10.1111/rda.12440, PMID: 25307982

[28] Fischer-Tenhagen C Tenhagen BA Heuwieser W. Short communication: ability of dogs to detect cows in estrus from sniffing saliva samples. J Dairy Sci. (2013) 96:1081–4. doi: 10.3168/jds.2012-5683, PMID: 23261382

[29] Fischer-Tenhagen C Wetterholm L Tenhagen B-A Heuwieser W. Training dogs on a scent platform for oestrus detection in cows. Appl Anim Behav Sci. (2011) 131:63–70. doi: 10.1016/j.applanim.2011.01.006

[30] Johnen D Heuwieser W Fischer-Tenhagen C. How to train a dog to detect cows in heat - training and success. Appl Anim Behav Sci. (2015) 171:39–46. doi: 10.1016/j.applanim.2015.08.019

[31] Dehnhard M. Mammal semiochemicals: understanding pheromones and signature mixtures for better zoo-animal husbandry and conservation. Int Zoo Yearbook. (2011) 45:55–79. doi: 10.1111/j.1748-1090.2010.00131.x

[32] Curry E Skogen M Roth T. Evaluation of an odour detection dog for non-invasive pregnancy diagnosis in polar bears (*Ursus maritimus*): considerations for training sniffer dogs for biomedical investigations in wildlife species. J Zoo Aquarium Res. (2021) 9:1–7. doi: 10.19227/jzar.v9i1.568

[33] Moser AY Bizo L Brown WY. Olfactory generalization in detector dogs. Animals (Basel). (2019) 9:702. doi: 10.3390/ani9090702 31546835 PMC6769875

[34] Hawkins C McCallum H Mooney N Jones M Holdsworth M. Sarcophilus harrisii. IUCN Red List Threat Species. (2019) 2008:eT40540A10331066. doi: 10.2305/IUCN.UK.2008.RLTS.T40540A10331066.en

[35] Woods GM Fox S Flies AS Tovar CD Jones M Hamede R. Two decades of the impact of Tasmanian devil facial tumor disease. Integr Comp Biol. (2018) 58:1043–54. doi: 10.1093/icb/icy118 30252058 PMC6927850

[36] Woods GM Lyons AB Bettiol SS. A devil of a transmissible cancer. Tropical Med Infect Dis. (2020) 5:50. doi: 10.3390/tropicalmed5020050, PMID: 32244613 PMC7345153

[37] Hogg C Hockley J. DPIPWE/ZAA husbandry guidelines for Tasmanian devil, *Sarcophilus harrisii*. Australia: Zoo and Aquarium Association (2013).

[38] Hesterman H Jones SM Schwarzenberger F. Reproductive endocrinology of the largest dasyurids: characterization of ovarian cycles by plasma and fecal steroid monitoring. Part I. the Tasmanian devil (*Sarcophilus harrisii*). Gen Comp Endocrinol. (2008) 155:234–44. doi: 10.1016/j.ygcen.2007.05.013, PMID: 17592734

[39] Hesterman H Jones SM Schwarzenberger F. Pouch appearance is a reliable indicator of the reproductive status in the Tasmanian devil and the spotted-tailed quoll. J Zool. (2008) 275:130–8. doi: 10.1111/j.1469-7998.2008.00419.x

[40] Keeley T O'Brien JK Fanson BG Masters K McGreevy PD. The reproductive cycle of the Tasmanian devil (*Sarcophilus harrisii*) and factors associated with reproductive success in captivity. Gen Comp Endocrinol. (2012) 176:182–91. doi: 10.1016/j.ygcen.2012.01.011, PMID: 22306283

[41] Owen D Pemberton D. Tasmanian devil: a unique and threatened animal. Australia: Allen & Unwin (2005).

[42] Reid-Wainscoat E. E. The behavioral significance of olfactory scent cues in the Tasmanian devil [Master's thesis]: UCLA. (2018).

[43] Guiler ER. Observations on the Tasmanian devil, *Sarcophilus harrisii* (Marsupialia: Dasyuridae) II. Reproduction, breeding and growth of pouch young. Aust J Zool. (1970) 18:63–70. doi: 10.1071/zo9700063

[44] Kybert N Prokop-Prigge K Otto CM Ramirez L Joffe E Tanyi J. Exploring ovarian cancer screening using a combined sensor approach: a pilot study. AIP Adv. (2020) 10:035213. doi: 10.1063/1.5144532

[45] Wilson C Campbell K Petzel Z Reeve C. Dogs can discriminate between human baseline and psychological stress condition odours. PloS One. (2022) 17:e0274143. doi: 10.1371/journal.pone.0274143, PMID: 36170254 PMC9518869

[46] Lit L Schweitzer JB Oberbauer AM. Handler beliefs affect scent detection dog outcomes. Anim Cogn. (2011) 14:387–94. doi: 10.1007/s10071-010-0373-2, PMID: 21225441 PMC3078300

[47] Johnen D Heuwieser W Fischer-Tenhagen C. An approach to identify bias in scent detection dog testing. Appl Anim Behav Sci. (2017) 189:1–12. doi: 10.1016/j.applanim.2017.01.001

[48] Fischer-Tenhagen C Johnen D Heuwieser W Becker R Schallschmidt K Nehls I. Odor perception by dogs: evaluating two training approaches for odor learning of sniffer dogs. Chem Senses. (2017) 42:435–41. doi: 10.1093/chemse/bjx020, PMID: 28444161

[49] Oh Y Kwon O Min SS Shin YB Oh MK Kim M. Multi-odor discrimination by rat sniffing for potential monitoring of lung cancer and diabetes. Sensors. (2021) 21:3696. doi: 10.3390/s21113696, PMID: 34073351 PMC8198436

[50] Lazarowski L Krichbaum S DeGreeff LE Simon A Singletary M Angle C. Methodological considerations in canine olfactory detection research. Frontiers in veterinary. Science. (2020) 7:7. doi: 10.3389/fvets.2020.00408, PMID: 32766296 PMC7379233

[51] Gazit I Goldblatt A Terkel J. The role of context specificity in learning: the effects of training context on explosives detection in dogs. Anim Cogn. (2005) 8:143–50. doi: 10.1007/s10071-004-0236-9, PMID: 15449101

[52] Porritt F Shapiro M Waggoner P Mitchell E Thomson T Nicklin S. Performance decline by search dogs in repetitive tasks, and mitigation strategies. Appl Anim Behav Sci. (2015) 166:112–22. doi: 10.1016/j.applanim.2015.02.013

[53] Slotnick BM Nigrosh BJ. Olfactory stimulus-control evaluated in a small animal olfactometer. Percept Mot Skills. (1974) 39:583–97. doi: 10.2466/pms.1974.39.1.583 4418626

[54] Sankar R Archunan G. Discrimination of bovine estrus-related odors by mice. J Ethol. (2005) 23:147–51. doi: 10.1007/s10164-004-0140-4

[55] Elliker KR Sommerville BA Broom DM Neal DE Armstrong S Williams HC. Key considerations for the experimental training and evaluation of cancer odour detection dogs: lessons learnt from a double-blind, controlled trial of prostate cancer detection. BMC Urol. (2014) 14:22. doi: 10.1186/1471-2490-14-22, PMID: 24575737 PMC3945616

[56] Waggoner P Lazarowski L Hutchings B Angle C Porritt F. Effects of learning an increasing number of odors on olfactory learning, memory and generalization in detection dogs. Appl Anim Behav Sci. (2022) 247:105568. doi: 10.1016/j.applanim.2022.105568

[57] Ghirlanda S Enquist M. A century of generalization. Anim Behav. (2003) 66:15–36. doi: 10.1006/anbe.2003.2174

[58] Mahoney A Weetjens BJ Cox C Beyene N Reither K Makingi G. Pouched rats' detection of tuberculosis in human sputum: comparison to culturing and polymerase chain reaction. Tuberculosis Res Treatment. (2012) 2012:716989. doi: 10.1155/2012/716989, PMID: 22848808 PMC3400328

[59] Mozuraitis R Buda V Kutra J Borg-Karlson AK. P- and m-cresols emitted from estrous urine are reliable volatile chemical markers of ovulation in mares. Anim Reprod Sci. (2012) 130:51–6. doi: 10.1016/j.anireprosci.2011.12.008, PMID: 22266248

[60] Mozuraitis R Buda V Borg-Karlson AK. Optimization of solid-phase microextraction sampling for analysis of volatile compounds emitted from oestrous urine of mares. Zeitschrift Fur Naturforschung Section C-a J Biosci. (2010) 65:127–33. doi: 10.1515/znc-2010-1-220, PMID: 20355332

[61] Sankarganesh D Ramachandran R Suriyakalaa U Saravanakumar VR Archunan G Akbarsha MA. Assessment of urinary volatile compounds and proteins in the female goat, *Capra hircus*: a pilot study to reveal potential indicators of oestrus. Reprod Domest Anim. (2019) 54:646–51. doi: 10.1111/rda.13407, PMID: 30659685

[62] Rajanarayanan S Archunan G. Identification of urinary sex pheromones in female buffaloes and their influence on bull reproductive behaviour. Res Vet Sci. (2011) 91:301–5. doi: 10.1016/j.rvsc.2010.12.005, PMID: 21316068

[63] Barman P Yadav MC Kumar H Meur SK Ghosh SK. Gas chromatographic-mass spectrometric analysis of chemical volatiles in buffalo (*Bubalus bubalis*) urine. Theriogenology. (2013) 80:654–8. doi: 10.1016/j.theriogenology.2013.06.012, PMID: 23876684

[64] Pluta K Jones PRH Drabinska N Ratcliffe N Carrington SD Lonergan P. The potential of volatile organic compound analysis in cervicovaginal mucus to predict estrus and ovulation in estrus-synchronized heifers. J Dairy Sci. (2021) 104:1087–98. doi: 10.3168/jds.2020-19024, PMID: 33189280

[65] Anitas O Goncu S. Comparison of different extraction solvents used in GC-MS analysis for detecting volatile odor compounds in heat cow sweat. Turkish J Vet Animal Sci. (2021) 45:411–8. doi: 10.3906/vet-2008-90

[66] Sankar R Archunan G. Identification of putative pheromones in bovine (*Bos taurus*) faeces in relation to estrus detection. Anim Reprod Sci. (2008) 103:149–53. doi: 10.1016/j.anireprosci.2007.04.014, PMID: 17507187

[67] Marneweck C. Jurgens A. Shrader A. M. Dung odours signal sex, age, territorial and oestrous state in white rhinos. *Proceedings of the Royal Society B-Biological Sciences*. (2017). 284. 10.1098/rspb.2016.2376 PMC5247502 28077775

